# A South African experience in applying the Adopt–Contextualise–Adapt framework to stroke rehabilitation clinical practice guidelines

**DOI:** 10.1186/s12961-019-0454-x

**Published:** 2019-06-06

**Authors:** Karen Grimmer, Quinette Louw, Janine M. Dizon, Sjan-Mari Brown, Dawn Ernstzen, Charles S. Wiysonge

**Affiliations:** 1 Division of Physiotherapy, Faculty of Medicine and Health Sciences, Stellenbosch University, Francie van Zijl Drive, Tygerberg, Cape Town, 7505 South Africa; 2 Clinical Teaching and Education Centre, College of Nursing and Health Sciences, Flinders University, Daw Park, 5041 South Australia; 3 International Centre for Allied Health Evidence, University of South Australia, City East Campus, Adelaide, 5000 Australia; 4 Hong Kong Polytechnic University, Hong Kong, Kowloon Hong Kong; 5 Cochrane South Africa, South African Medical Research Council, Francie van Zijl Drive, Parow Valley, Cape Town, 7505 South Africa

## Abstract

**Background:**

Clinical practice guideline (CPG) activity has escalated internationally in the last 20 years, leading to increasingly sophisticated methods for CPG developers and implementers. Despite this, there remains a lack of practical support for end-users in terms of effectively and efficiently implementing CPG recommendations into local practice. This paper describes South African experiences in implementing international CPG recommendations for best practice stroke rehabilitation into local contexts, using a purpose-build approach.

**Methods:**

Composite recommendations were synthesised from 16 international CPGs to address end-user questions about best practice rehabilitation for South African stroke survivors. End-user representatives on the project team included methodologists, policy-makers, clinicians, managers, educators, researchers and stroke survivors. The Adopt–Contextualise–Adapt model was applied as a decision-guide to streamline discussions on endorsement and development of implementation strategies. Where recommendations required contextualisation to address local barriers before they could be effectively implemented, prompts were provided to identify barriers and possible solutions. Where recommendations could not be implemented without additional local evidence (adaptation), options were identified to establish new evidence.

**Findings:**

The structured implementation process was efficient in terms of time, effort, resources and problem solving. The process empowered the project team to make practical decisions about local uptake of international recommendations, develop local implementation strategies, and determine who was responsible, for what and when. Different implementation strategies for the same recommendation were identified for different settings, to address different barriers.

**Conclusion:**

The South African evidence translation experience could be useful for evidence implementers in other countries, when translating CPG recommendations developed elsewhere, into local practice.

## Background

WHO has called for innovative, evidence-based rehabilitation action to deal with the increasing burden of disability internationally [1] and has also noted that rehabilitation services are often not accessible or ideal in many low- and middle-income countries (LMICs) due to scarce resources and heavy disease burdens [1, 2]. The World Health Assembly Resolution on Disability determined that rehabilitation can contribute to reducing poverty through improving functioning, activity levels and societal participation [3]. Moreover, the lack of effective rehabilitation can adversely affect health, evidenced by increased rates of complications and healthcare utilisation [3]. South Africa has a huge and complex burden of disease [1–3]. Putting best rehabilitation evidence into widespread practice is urgent to ensure that all South Africans with disability can access equitable, effective and timely rehabilitation.

Reflecting the increasing prevalence of stroke worldwide, and its impact on individuals, families and society, there has been significant international research activity resulting in many clinical practice guidelines (CPGs). Treweek et al. described CPGs as “*a convenient way of packaging evidence and presenting recommendations to healthcare decision makers*” [4]. Our recent search for freely available CPGs for stroke rehabilitation, published from 2010 onwards, identified 16 CPGs of mostly moderate-to-good methodological quality, from the United Kingdom (*n* = 2), United States of America (*n* = 5), Australia (*n* = 5) and one each from Canada, Malaysia, New Zealand and South Africa [5]. These CPGs are listed in Box 1. The South African stroke CPG was 7 years old, dealing mostly with medical stroke care and providing few rehabilitation recommendations.

There were many consistent recommendations across the CPGs, and the seven CPGs published between 2015 and 2017 mostly cited the same evidence base [5]. Thus, to provide up-to-date guidance for stroke rehabilitation in South Africa, there appeared to be no need to reproduce the evidence-seeking and synthesis steps to write another CPG [4]. Rather, it was more important to focus on implementing existing international recommendations into South African practice. Our approach built on Eisenberg’s notion of “*globalizing the evidence but localizing the decision*” ([6], p. 166). We developed novel methods, firstly to synthesise recommendations extracted from the international CPGs that were relevant to South African stroke rehabilitation questions, and secondly to describe the composite evidence base underpinning each synthesised recommendation [5]. For the first method, we applied a qualitative approach to interrogate the intent of the wording of relevant CPG recommendations, and then we drafted composite recommendations that reflected the essence of the individual recommendations. For the second method, we amalgamated the strength and consistency of the underpinning evidence, weighted by the number, methodological quality and currency of relevant CPGs [5]. This was expressed as one, two or three ticks for positive recommendations (‘Do’), one, two or three crosses for negative recommendations (‘Don’t do’), as well as indicators for insufficient or inconsistent evidence [5]. Examples of this system are provided in Table 2.

CPGs generally provide recommendations about ‘what to do’, based on intervention studies [4]. Evidence regarding how services should best be provided (the ‘who, how, when, how much, why’ questions) are usually reported in CPGs as Consensus or Practice Points, written by local clinical and policy experts. This reflects the often-scant research about best practice service delivery, and the essential link between service delivery recommendations and local contexts [1–3, 5, 7]. WHO has proposed characteristics of good quality service delivery required to effectively implement CPG recommendations [8]. These characteristics speak to inputs (such as workforce, service comprehensiveness, resources, continuity, coordination, accountability) and outputs (care processes and health outcomes, measured as person-centredness, efficiency, equality, equity, access, timeliness and effectiveness). Getting local service delivery mechanisms correct is the nub of effective implementation [4, 5, 7–9].

In South Africa, stroke is a leading cause of disability among adults of all ages due to co-morbidities such as HIV/AIDS and tuberculosis [1, 10, 11]. Stroke can have long-term social and financial implications, particularly if rehabilitation is sub-optimal. South African stroke rehabilitation occurs in a range of settings, from quaternary metropolitan hospitals to remote community primary healthcare (PHC) settings, which are often understaffed and under-resourced [10, 11, 21, 22]. Providing current best-evidenced guidance in stroke rehabilitation, contextualised to South African healthcare providers, patients and settings, offers a way to improve care within scarce resources [9–15].

There is increasing research about implementing evidence in LMICs [7, 12–17]. “*We know what we have to do, but we don’t know how to do it*” was noted by Ridde as a “*recurring comment among global health actors*” [7]. Research into evidence implementation and uptake in LMICs has largely been regarding knowledge translation into policy, identifying gaps between research and end-user/stakeholder needs for guidance [14–17]. It also appears that the challenges of evidence implementation into clinical practice in LMICs are yet to be fully identified or addressed [14, 15]. Alonge et al. [12] noted that future LMIC implementation research should more clearly describe the problems, strategies, contexts, concepts, methods and outcomes of implementing evidence into often complex and non-replicable situations to produce better information about what works or does not work. Research among South African allied health professionals (who largely provide stroke rehabilitation) identified many barriers when applying CPGs in PHC. Some barriers were similar to those in high-income countries (HICs) (e.g. lack of training, organisational support, access to information, resources and recognition of effort [18–20]). However, there are specific LMIC barriers that have attracted less research attention, such as economic and geographic diversity in how healthcare is delivered, financial and physical constraints on patients’ access to care, high patient to therapist workload, variably skilled workforce, poor patient health literacy, and influence of religion, traditional healers and cultural beliefs [10, 11, 21, 22].

An increasing volume of literature has been published since 1990 on frameworks, models and strategies to support behaviour change, evidence uptake, transfer of evidence to practice and evidence implementation. This underscores the intellectual investment of many international researchers in making sense of the complexities of putting evidence into effective, sustainable practice. This interest was flagged in 2001, when Grimshaw et al. [23] summarise 41 systematic reviews on effective strategies of putting evidence into practice. They concluded then, that the best approach to evidence implementation was individual and fitted to circumstance. They recommended steps for successful evidence implementation as “*a) prepare well; b) involve the relevant people; c) develop a proposal for change that is evidence based, feasible, and attractive; d) study the main difficulties in achieving the change, and select a set of strategies and measures at different levels linked to that problem; of course, within your budget and possibilities; e) define indicators for measurement of success and monitor progress continuously or at regular intervals; and, finally, f) enjoy working on making patients’ care more effective, efficient, safe, and friendly*” [23].

Since 2001, there have been many more strategies, models and frameworks proposed for effective behaviour change, knowledge translation and/or evidence uptake at organisational, policy, environmental, educational and individual levels. Nilsen [9] summarised implementation research in 2015, and proposed five groupings (process models, determinant frameworks, classic theories, implementation theories and evaluation frameworks). However, he also noted that “[m]*ost determinant frameworks provide limited “how-to” support for carrying out implementation endeavours since the determinants usually are too generic to provide sufficient detail for guiding an implementation process*” ([9], p. 53)*.* Examples of models, frameworks and strategies are provided in Box 2 [24–37].

We mapped our understanding of the complexity of South African rehabilitation [5, 7, 10, 11, 16] against the approaches in Box 2 [24–37], but there was no immediately relevant method that supported evidence implementation into the complexity of South African rehabilitation service delivery [7, 8, 10–15].

### What is known about the topic

Reflecting the high resource and time costs of developing de novo CPGs, there is increasing interest in transferring CPG recommendations developed in one setting to another. Two approaches have been proposed for transferring CPG recommendations between HICs (ADAPTE [38, 39] (the ADAPTE Collaboration is a partnership between two independent groups focusing on guideline adaptation – ADAPTE group and the Practice Guideline Evaluation and Adaptation Cycle group) and GRADE-ADOLOPMENT [40] (a new term added to the clinical epidemiology Lexicon), although neither has been tested in LMICs. There is only one approach that we know of to have transferred HIC CPG recommendations into a LMIC context, without rebuilding the underpinning evidence base – the Filipino Contextualisation approach (2011–2012), developed by clinicians and researchers who transferred recommendations from multiple HIC CPGs for stroke and lower back pain to Filipino contexts [41]. To do this, typical Filipino patient pathways were drafted for these conditions, HIC CPG recommendations were mapped to the pathways, and local barriers to evidence implementation were identified. Strategies were designed to address local barriers to evidence uptake, which considered workforce, the hierarchical structure of the Filipino healthcare system, clinician training, communication, geographical access to care, culture and health literacy [41].

The Filipino Contextualisation approach was expanded in Project SAGE, a South African research project (2014–2017) which investigated the use of CPGs in South African PHC [42, 43]. An output of Project SAGE was a three-tiered model of locally implementable CPGs, where Tier 1 is the international evidence base (‘what to do’), Tier 2 is local expert input placing context around recommendations (and identifying implementation challenges), and Tier 3 comprises contextually relevant end-user documents to support local evidence uptake into practice [44] (‘how to do it in the local setting’).

The Adopt–Contextualise–Adapt (ACA) approach was developed to run parallel with the Project SAGE CPG Tiers model [44], and underpin practical and efficient transfer of Tier 1 CPG recommendations into South African PHC settings [45]. The ACA approach proposes strategies to bridge evidence–local implementation gaps, and address local implementation barriers [9–18]. The ACA model is underpinned by the notion that global recommendations from HIC CPGs can be implemented in LMIC settings by adding contextually relevant ‘how to do it’ information [6]. The ACA model is flexible, and is applied to each recommendation. Some recommendations may be readily transferred from one setting to another (irrespective of HIC or LMIC status) (‘adopted’). Other recommendations however, may require ‘contextualisation’ before they can be effectively transferred from HIC to LMIC settings. Contextualisation is when locally relevant strategies are developed to overcome local barriers to effective local implementation of recommendations extracted from CPGs developed elsewhere. This process makes no change to recommendation wording. In other circumstances, recommendations from HIC CPGs may simply not be accurate, practical or effective in LMICs because of culture, disease epidemiology or service delivery barriers (e.g. environment, organisation, workforce, training) [9–18]. Thus, recommendations may need to be ‘adapted’ (changed) by adding new evidence (derived from local research or expert opinion) before they can be operationalised locally. This might mean, for instance, recommending a different treatment to obtain the same effect when the recommended treatment is unavailable, unaffordable or even ineffective in the local population [45]. We contend that the terms ‘adoption’, ‘contextualisation’ and ‘adaptation’ reflect different evidence transfer processes [45], and that use of the term ‘adaptation’ to describe the transfer between HICs [38–40] of unchanged recommendations layered with local strategies is, in fact, contextualisation.

### What new knowledge does this paper contribute

This paper describes experiences in addressing the ‘how to do it’ to implement stroke rehabilitation recommendations from CPGs developed elsewhere into South African contexts. These experiences may assist other LMICs wishing to implement HIC CPG recommendations into local practice.

## Methods

### Funding and scope

Seed funding was provided by Stellenbosch University and the WHO Alliance for Health Policy and Systems Research in February 2017, to produce evidence-based guidance for stroke rehabilitation relevant to South Africa. The draft guidance document was required to be completed by December 2017 (a 10-month project). There was neither time nor resources to ‘recreate the wheel’ of evidence about ‘what to do’, nor, in fact, was there any need for this given the wealth of evidence-based guidance already available internationally for effective stroke rehabilitation [5]. The decision was thus made by the project team to focus on implementation, and build on a compilation of available international evidence on stroke rehabilitation, rather that write a de novo South African CPG for rehabilitation after stroke.

The project team identified 38 questions for which answers (recommendations) were sought from international stroke rehabilitation CPGs. Using the new methods for evidence synthesis [5], 78 composite recommendations were produced and reported as the South African-contextualised stroke rehabilitation CPG [5]. However, simply producing evidence statements does not ensure their uptake by end-users, particularly in a country where health service delivery is complex [7, 9–18]. Thus, our challenge was to develop specific implementation plans for each composite recommendation to ensure its relevance to, and timely uptake in, local South African practice.

We sought guidance from the implementation literature by considering commonly cited frameworks, theories and models (Box 2). We also considered the summaries of gaps in the implementation research literature [7, 9, 15] and realised that there was no immediately applicable approach. To keep the process as simple as possible, the team identified that the first six elements of Grimshaw’s advice were the most relevant to guide our work [23], namely (1) prepare well, (2) invite the relevant people, (3) develop a proposal for change that is evidence based, feasible and attractive, (4) study the main difficulties in achieving the change, (5) select a set of strategies and measures at different levels linked to that problem, within your budget and possibilities, and (6) define indicators for measurement of success and monitor progress continuously or at regular intervals. The ways in which we acted on this advice are outlined below.

### Prepare well

The scope and purpose of the funding for the new South African-relevant stroke rehabilitation CPG was to address an urgent national need to improve access, equity and quality of rehabilitation for people with stroke. We believed that we could provide the vehicle for ‘how to do it’ by developing locally relevant implementation plans for recommendations complied from 16 international CPGs for stroke rehabilitation [5].

### Involve the relevant people

A national team comprising methodologists, stroke researchers, educators, national and provincial policy-makers, facility managers, expert clinicians and stroke survivors was established. Team members brought broad end-user perspectives and knowledge to the table to assist with barrier identification and solutions. Their engagement and input reflected Tier 2 of the CPG Tiers model [44]. The team met six times over 4 months, with clear purposes for each meeting, including (1) developing typical South African stroke patient pathways that described the points of entry to, and exit from, the South African healthcare system, depending on where patients live, local healthcare options, and their capacity to pay; (2) determining questions along the patient pathways that required evidence-based answers (recommendations) [5, 41]; (3) agreeing on wording of composite recommendations when multiple CPGs provided recommendations relevant to the same question; (4) participating in barrier identification and finding solutions to enhance evidence implementation [45] (Tiers 2 and 3 of the Project SAGE CPG model [44]); and (5) drafting implementation plans for each recommendation [7, 9, 45].

### Develop a proposal for change that is evidence based, feasible and attractive

The project’s implementation focus and the ACA model made sense to the project team [5, 44, 45]. The team also accepted that there was no need to create yet another stroke rehabilitation evidence base, because of the large volume of evidence statements available internationally. The team also accepted the challenge of producing practical implementation plans that could make a sustainable difference to SA stroke rehabilitation practices nationally, irrespective of the healthcare setting. Initially, for training, efficiency and quick successes, the project team considered only those recommendations underpinned by strong evidence. Once team members were more comfortable with the process, they then considered the less well-evidenced recommendations.

### Study the main difficulties in achieving the change

**Fig. 1:**
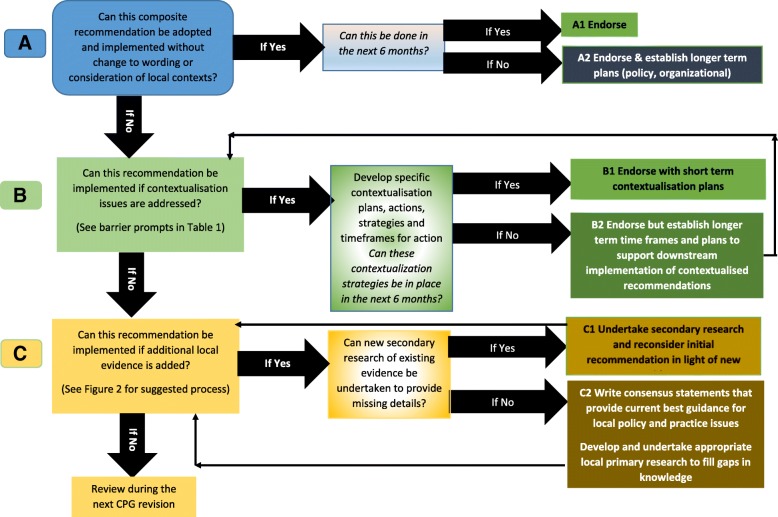
Adopt–Contextualise–Adapt (ACA) decision-making process [45]

The methodologists on the team developed a solution-focused algorithm to operationalise the ACA approach efficiently (Fig. 1). We labelled this process ‘endorsement’, which differed in meaning from its use in the CPG development literature [47]. This refers to ratification of an overall CPG by stakeholders (such as Government, professional associations, insurers, etc.) [47]. In our ACA approach, however, the term ‘endorsement’ follows its prior use in the Philippines, reflecting ‘sign-off’ on the wording of composite recommendations, the underpinning evidence base and the recommendation-specific implementation decisions [41].

The first endorsement process (Fig. 1a) was to identify adoptable recommendations. All recommendations were scanned, and those which we believed could be readily implemented ‘as is’ (without change to wording or the need to address local barriers) were identified. We then considered whether different South African healthcare settings were equally likely to be able to action the potentially adoptable recommendations. This was particularly important given the diversity of South African healthcare settings in which stroke rehabilitation occurs (differently sized, resourced and staffed hospital and community centres in metropolitan, urban, regional, rural and remote areas). The timeframe was determined over which adoption of each recommendation might occur, and broad strategies were determined to support efficient evidence uptake across different settings.

The second endorsement process (Fig. 1b) was to identify recommendations which could be implemented after local contextual barriers were addressed. Building on the WHO characteristics of health service quality (‘who, how, when, how much, why’), the team’s knowledge of local circumstances [10, 11, 21, 22] and the Filipino barriers perceived as constraining evidence uptake, a list of potential South African barriers was produced to prompt discussions (Table 1) [8, 41]. We recognised that different healthcare settings in South Africa potentially experience different barriers to evidence uptake, and thus different contextualisation approaches may be required. This reflected the Eisenberg notion of “*globalise evidence, localize (implementation) decisions*” [6]. Thus, barriers (and solutions) to implementing a recommendation may need to be specific-to-setting (for instance, in a rural PHC setting or a metropolitan hospital). This approach was underpinned by the Eisenberg notion of local applications of a global evidence base [6].

**Table 1:** Broad barrier prompts for contextualisation discussions

	What is required to effect change?	In minimum standard of care	In higher standard of care	Training required? If so what, and for whom?
Organisation • Resources • Type of workforce	Responses should identify and address specific local issues	In the least resourced environment for care provision (eg community clinics)	In a well resourced environment for care provision (eg metropolitan tertiary hospital)	Who requires additional training to implement each recommendation? What training is required, and how should it be delivered?
Service delivery • Legislative responsibilities/constraints • Availability of workforce	Responses should identify and address specific local issues	In the least resourced environment for care provision (eg community clinics)	In a well resourced environment for care provision (eg metropolitan tertiary hospital)	Who requires additional training to implement each recommendation? What training is required, and how should it be delivered?
Communication • People • Resources (phone, internet, fax)	Responses should identify and address specific local issues	In the least resourced environment for care provision (eg community clinics)	In a well resourced environment for care provision (eg metropolitan tertiary hospital)	Who requires additional training to implement each recommendation? What training is required, and how should it be delivered?
Clinical care • Availability of workforce • Type of workforce • Capacity of workforce • Available equipment • Other available resources	Responses should identify and address specific local issues	In the least resourced environment for care provision (eg community clinics)	In a well resourced environment for care provision (eg metropolitan tertiary hospital)	Who requires additional training to implement each recommendation? What training is required, and how should it be delivered?

**Fig. 2:**
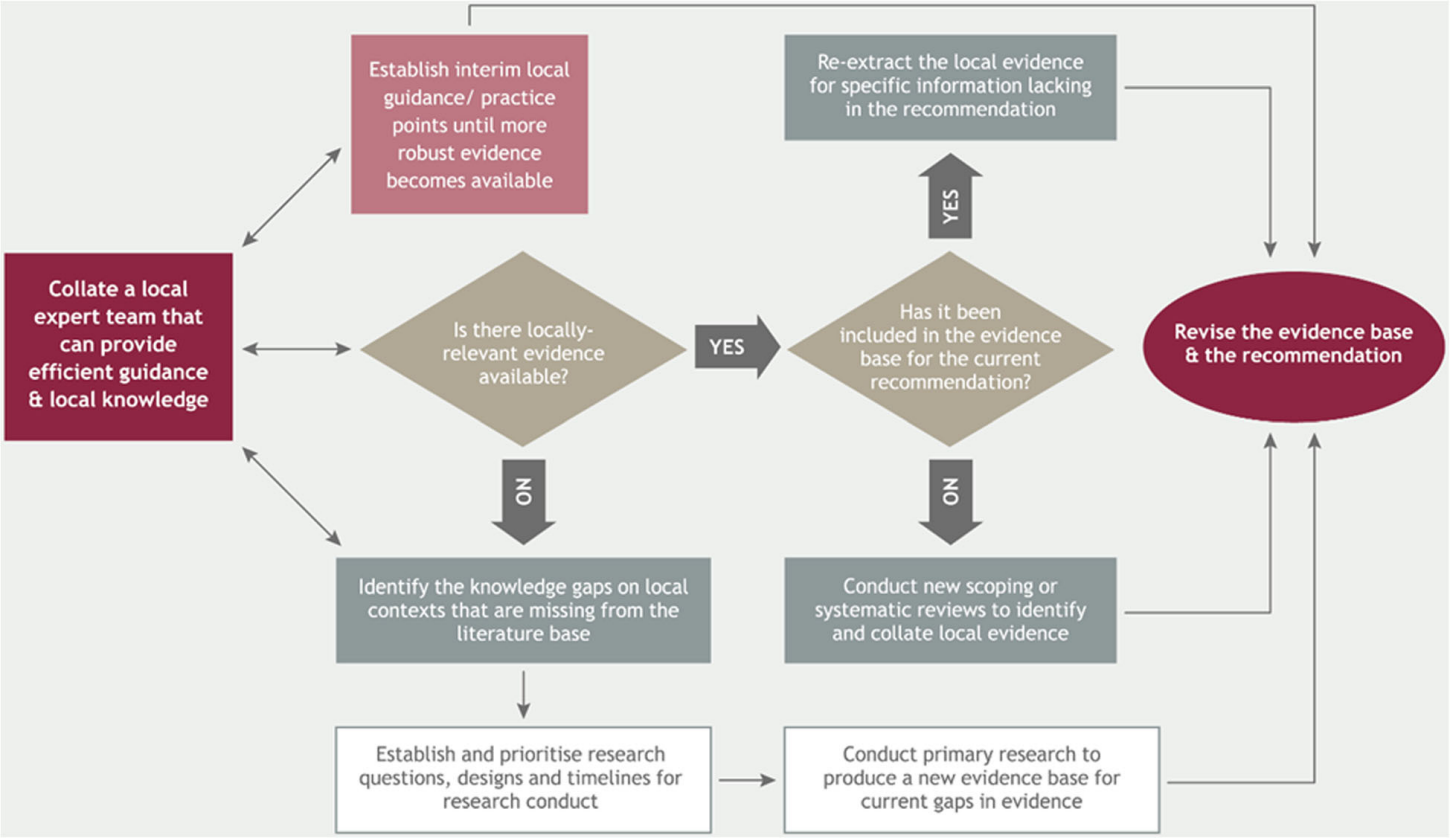
Suggested processes to address the need for additional evidence for the recommendations requiring adaptation

The third endorsement process (Fig. 1c) applied to those recommendations which required ‘adaption’ before they could be effectively implemented in South Africa. These recommendations were not relevant to South African funding models, legislation, service provision, local healthcare providers, local communities, stroke patient needs and/or local disease aetiology; they thus required additional evidence (expert input and/or local research) to make them locally implementable. We developed a discussion guide to assist in determining where, and how, additional evidence might be sourced (Fig. 2).

### Select a set of strategies and measures at different levels linked to that problem, within your budget and possibilities

Discussions involved different configurations of end-users (e.g. within the same group (just clinicians or just policy-makers) as well as in mixed groups (clinicians, managers, researchers, stroke survivors)). Discussions were based on the ACA decision-making algorithm (Fig. 1) and the barrier prompts (Table 1). Participants were encouraged to speak freely about their experiences in their settings and to seek practical solutions (rather than discard recommendations on the basis that they cannot be implemented ‘right now’). The benchmark was an A1 endorsement (recommendation could be implemented ‘as is’ within the next 6 months) and end-users were encouraged to identify specific reasons as to why this might not occur for each recommendation. The timeframe of 6 months was determined by the team as being sufficiently long for action to occur, but short enough to focus people’s energies and keep the implementation challenge ‘on the agenda’, whilst being within the current budget cycle. This approach proved to be a powerful tool to give a voice to end-users, who were advocates for their local environment and patients, and were encouraged to articulate solutions such as ‘*if only we could …*. ’, or ‘*what about doing …*. ’?, or ‘*how long would that take us?*’.

### Define indicators for measurement of success and monitor progress continuously or at regular intervals

For each endorsement, the project team discussed auditable outcome measures that might demonstrate the success of implementation plans.

## Results

### Endorsement outcomes

**Fig. 3:**
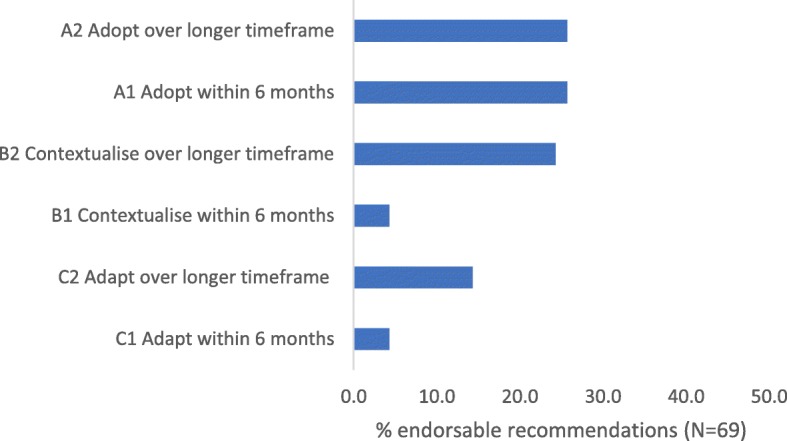
Percentage of total endorsable recommendations for each endorsement category

Of the 78 composite recommendations derived from the included CPGs, 13 had insufficient or equivocal evidence, and the team did not pursue endorsements for these (leaving 65 endorsable recommendations). Of these, four attracted two endorsements each (most commonly adoption (A1 or A2) coupled with recognition that additional evidence was needed (C1 or C2)). The most common endorsements overall were A1 (Adopt within the next 6 months) and A2 (Adopt over the longer term) (25.1%) and B1 (Contextualise within the next 6 months) (24.6%). Figure 3 reports the percentage of each endorsement category of the total number of endorsement options (*N* = 69). The A1 endorsements are provided in Box 3 (Prof Louw can be contacted for other recommendations). Exemplar endorsed recommendations, their composite evidence strength and implementation strategies are provided in Table 2.

**Table 2:** Examples of recommendations, Adopt–Contextualise–Adapt decisions (Fig. 1) and implementation plans [5]

	Evidence strength^a,b^	Recommendations	Endorsement	Implementation plans
1.	✓✓✓	There are consistent strong recommendations that people who suffer from stroke should be seen by a multidisciplinary/inter-professional/interdisciplinary stroke team for medical and rehabilitation assessment and management. The team consists of doctors, nurses, physiotherapists, occupational therapists, speech language therapists, social workers, dieticians, clinical neuropsychologists/clinical psychologists	B2	Increase the rehabilitation workforce (requiring at least 5 years for students to graduate from new university programmes) Increase funding to support a multidisciplinary stroke workforce (requiring at least 2 years to lobby, plan and train)
2.	✓✓	There are consistent suggestions that all members of the multidisciplinary team should have specialised training in stroke care and recovery	A2	Increase the amount of tertiary training in rehabilitation in medical, nursing and allied health programmes (requiring a 2–3 year timeframe)
3.	✓✓✓	There are consistent strong recommendations that all patients who suffer from stroke should have access to specialist stroke service units with multidisciplinary team as early as the hyperacute–acute stages of stroke and up to discharge	B2	Same solutions as for 1, but including changing opinions of hospital administrators, policy-makers and funders about the value of specialist stroke service units
4.	✓✓✓	There are consistent strong recommendations that rehabilitation should commence in the acute setting as soon as the person with stroke is medically safe and/or able to participate	A1	There are no barriers to this, but it may be important to educate medical doctors to refer stroke suffers as quickly as possible for rehabilitation
5.	✓✓	There are consistent suggestions that a standard set of assessment tools should be used to assess rehabilitation needs throughout the patient journey; these should be valid, sensitive to detect change, simple to use and, if required, apply standard protocols to assist more complex assessment	C2	Current international assessment tools require further evaluation for local contexts and fit to South African rehabilitation settings Local tools dealing with local outcome issues may need to be developed

^a^✓✓✓ is assigned when the composite recommendation is underpinned by three or more CPG recommendations that have a high strength of the body of evidence grading, and provide positive consistent recommendations [5]

^b^✓✓ is assigned when the composite recommendation is underpinned by three or more CPG recommendations that are supported by moderate strength of the body of evidence gradings, and provide positive consistent recommendations [5]

## Discussion

This is only the second LMIC research project that we know of that has taken planned steps to progress evidence statements to implementation actions (the first occurring in the Philippines [41]). In hindsight, our team could have been overwhelmed by the enormity of the task, however the structured decision-making processes were supportive and effective in keeping discussions focused and efficient. The project team was regularly reminded at each meeting of Grol and Grimshaw’s advice to “*enjoy working on making patients’ care more effective, efficient, safe, and friendly*” ([23], p. 1229). Not only were site-specific solutions identified for a range of barriers, but timeframes were established within which to action solutions and achieve results (short (6 months) or longer-term). The project team appeared to enjoy the exercise of translating evidence to action, and felt empowered to consider implementation because the task of developing the evidence base had been removed. Solutions to problems were drafted for different healthcare settings (e.g. basic levels of care (primary and secondary sectors) and in more advanced care settings (e.g. tertiary and quaternary sectors)). This approach encouraged end-users to embrace the notion of locally specific implementation of evidence, irrespective of healthcare setting, training or experience [6].

A key learning from this project was to be open minded and consider every composite recommendation as potentially relevant to, and achievable across South African rehabilitation contexts. This notion was important to create a positive, problem-solving mindset around implementing best practice for rehabilitating people with stroke, right from acute care to community re-integration. The objectives of this project appeared to tap into sensitivities around implementation [7, 9, 12–18]. For many recommendations, initial reactions from many team members were to dismiss them with statements such as “*we can’t do that here*” or “*that is not feasible*”. However, by providing step-by-step guidance to consider each recommendation in context, solutions that applied the WHO quality service delivery outcomes of equity, person-centredness, effectiveness, efficiency, timeliness and access prevailed, and solutions were quick to emerge [1–3, 8].

Consider the recommendation to ‘Establish best practice multidisciplinary allied health stroke rehabilitation teams at all points of entry to the healthcare system’ in Table 2. Specialist multidisciplinary stroke teams working in dedicated acute stroke units have been shown internationally to significantly decrease mortality and length of stay (irrespective of type of stroke and age of patient), significantly improve function, quality of life and return to employment, and reduce residual disability in the short and long term [48]. Our challenge was that, currently, there are few dedicated acute stroke units or specialist multidisciplinary stroke care teams in South African hospitals. This knowledge could only have come from our team (Tier 2 local experts) because there was no South African research on gaps between current and international practices in stroke rehabilitation. Whilst our team recognised the potential value of implementing this recommendation, it also recognised the many constraints to constructing, training and supporting multidisciplinary teams on dedicated South African stroke wards. On this basis, the endorsement for this recommendation was B2, which is ‘Endorse but establish longer term time frames and plans to support downstream implementation of contextualised recommendations’. Possible solutions to implement multidisciplinary stroke teams on dedicated stroke wards were proposed to the policy-maker team members, with a view to progressing policy change and funding to establish a small number of exemplar stroke units as soon as possible, and evaluating them for cost, process and health outcomes.

For some recommendations, indicators of success were obvious; however, for others, indicators could not be set until the recommendation is close to being achieved. Again, considering the recommendations regarding specialist multidisciplinary stroke teams providing care on dedicated acute stroke units [48] (Table 2), 5-year national process indicators could be (1) the number of South African hospitals with specialist multidisciplinary stroke teams and dedicated acute stroke units in place; (2) the processes by which multidisciplinary rehabilitation plans are made, and actioned; (3) patient and family engagement in rehabilitation plans; (4) the number of universities offering multidisciplinary stroke rehabilitation specialist training; (5) the number of students enrolling in tertiary allied health training programmes in rehabilitation streams; and (6) availability of, and access to, specialist training to improve multidisciplinary rehabilitation care.

Outcome indicators could be hospital stroke survival rates; length of stay for acute stroke patients; streamlined referral for community rehabilitation after hospital discharge; and residual disability and employment post discharge.

The consultative process of identifying barriers for each recommendation and finding solutions to barriers proved to be relatively straightforward, even for inexperienced project team members. By sensibly identifying and deconstructing local implementation barriers, focusing on improved patient access, outcomes and quality of care, and drawing on the problem-solving skills of the project team, it is likely that the quality of stroke rehabilitation in South Africa will significantly improve within the next 5 years.

The ACA decision-making algorithm, and associated support documents, appear to provide a way of efficiently, and practically, translating recommendations from CPGs developed in HICs, to an LMIC with enormous disease burden, limited resources, and complex and variable service delivery options. The WHO service quality process and outcome characteristics [8], the Filipino Contextualisation approach [41] and the composite international evidence base [5] provided frameworks to guide discussions. Our experiences with the South African stroke rehabilitation CPG suggest that LMICs could be advised to focus on implementing existing good quality evidence from CPGs developed elsewhere, rather than recreating evidence-seeking activities [12–18]. The process of CPG writing is highly evolved in HICs, particularly in continual updating with new literature, and technology to support bedside decision-making [38–40, 46, 47]. However, for CPGs to be convenient packages of evidence for LMICs [4], implementation needs to be practical [6, 23], draw on local strengths and knowledge [7, 10–18, 46], and develop contextually relevant theories [7, 9–18].

## Conclusion

Our experiences were largely positive, and what we learnt offers a way forward not only for South African CPGs to be efficiently written for other conditions, but also for other LMICs to improve healthcare. Our team members are now change champions, who are empowered to disseminate practical approaches to evidence dissemination within their profession and their workplace. We anticipate that increased awareness of CPG recommendations, and improved capacity to identify and solve local implementation barriers will be reflected in reduced mortality, disease burden and length of hospital stay, as well as improved function, quality of life and community participation for South African stroke survivors. Thus, the impact of applying the ACA approach and its associated decision-making processes to one prevalent and potentially debilitating condition in one LMIC could significantly impact on its economy, and provide a blueprint for considering best practice care for other problematic health conditions.

**Box 1** Included CPGs Canadian Stroke Guidelines (2015) http://www.strokebestpractices.ca/acute-stroke-management/

Malaysian Stroke Guideline (2016) http://www.acadmed.org.my/index.cfm?menuid = 67#Cardiovascular_Disease

National Institute for Health and Clinical Excellence (2013) https://www.nice.org.uk/guidance/cg162

Royal College of Physicians (2012) https://www.rcplondon.ac.uk/guidelines-policy/stroke-guidelines

American Heart Association/American Stroke Association (Rehabilitation Guideline) (2015) https://www.ahajournals.org/doi/pdf/10.1161/STR.0000000000000098

American Occupational Therapy Association (sourced through Guidelines Clearing House AHRQ) (2013) https://www.guidelinecentral.com/summaries/occupational-therapy-practice-guidelines-for-adults-with-stroke/

Department of Defense, Veterans Association Management of Stroke Guidelines (2010) https://www.rehab.research.va.gov/jour/10/479/pdf/vadodcliniaclguidlines479.pdf

New Zealand Guidelines Group (2010) https://www.health.govt.nz/publication/new-zealand-clinical-guidelines-stroke-management-2010

NSW Agency for Clinical Innovation (2016) https://www.aci.health.nsw.gov.au/networks/stroke

South Australian Dept Health Stroke Network (2017) https://www.sahealth.sa.gov.au/wps/wcm/connect/ae53950047066243b403fc22d29d99f6/Clinical+Guideline_Stroke+Management_Proceudres+and+Protocols_final+Oct14pdf?MOD=AJPERES&CACHEID=ae53950047066243b403fc22d29d99f6&CACHE=NONE

Guidelines Clearing House https://www.guidelinecentral.com/summaries/stroke-diagnosis-and-initial-management-of-acute-stroke-and-transient-ischaemic-attack-tia/

Scottish Intercollegiate Guidelines Network

- Dysphasia (2010) https://www.sign.ac.uk/assets/sign119.pdf
- Rehabilitation (2010) https://www.sign.ac.uk/sign-118-management-of-patients-with-stroke-rehabilitation,-prevention-and-management-of-complicati.html

Australian Council on Safety and Quality in Health Care (2015) https://www.safetyandquality.gov.au/our-work/clinical-care-standards/acute-stroke-clinical-care-standard/

Australian Stroke Foundation (2017) https://strokefoundation.org.au/What-we-do/Treatment-programs/Clinical-guidelines

South African Stroke Society (2010) https://www.ncbi.nlm.nih.gov/pubmed/21081029

**Box 2** Examples of frameworks, models and strategies for aspects of implementation Ottawa Model of Research Use [24]

Diffusion of innovations in service organisations [25]

Domains of behaviour change [26]

The OFF theory [27]

Pipeline of knowledge uptake [28]

The Knowledge to Action Model [29]

TCU Organisational Readiness for Change [30]

Translating Evidence into Practice Model [31]

Behaviour Change Wheel [32]

Consolidated Framework for Implementation Research [33]

Steps for developing a theory-informed intervention [34]

Conceptual framework for planning and improving evidence-based practices [35]

Context and Implementation of Complex Interventions framework [36]

Ontology of Behaviour Change Interventions [37]

Box 3 Recommendations with A1 endorsements (adoptable in South African rehabilitation sites within 6 months) 4. There are consistent strong recommendations that the rehabilitation processes should commence in the acute setting as soon as the person with stroke is medically safe and/or able to participate.

8. There are consistent suggestions that a standardised clinical assessment should be applied by a professional skilled in the management of dysphagia (currently speech and language therapists).

10. There are consistent suggestions that education should be made available to all healthcare providers about adverse events following stroke.

11. There are consistent suggestions that all patients with a stroke should be mobilised as early as possible, to lessen likelihood of complications such as pneumonia, deep vein thrombosis, pulmonary embolism and pressure sores.

12. There are consistent suggestions that patients with mild and moderate stroke should be provided with frequent, short activity sessions.

13. There are consistent strong recommendations against the routine use of splints or prolonged positioning of upper or lower limb muscles in a lengthened position (stretch) for stroke survivors who are at risk of developing contracture.

42. There are consistent suggestions that comprehensive assessment of rehabilitation needs should include:

- Previous functional abilities;
- Impairment of psychological functioning (cognitive, emotional) and communication;
- Impairment of body functions, including pain/orientation;
- Activity limitations and participation restrictions, e.g. positioning, moving, transfer and handling;
- Swallowing;
- Pressure area risk;
- Continence;
- Nutritional status and hydration;
- Environmental factors (social, physical and cultural).

48. There are consistent strong recommendations that physiotherapists, occupational therapists, speech and language therapists, and dieticians bring specific competencies and skills to patient assessment and rehabilitation planning as members of the multidisciplinary team. They operate most effectively when sharing assessment and rehabilitation tasks, and communicating findings verbally and in written form in patient notes.

50. There are consistent suggestions that treatment decisions should be clearly documented.

51. There are consistent suggestions that progression of rehabilitation programmes should be documented, including reason for progression and patient responses.

53. There are consistent suggestions that progress reports on interventions and outcomes should be communicated regularly within the team, and to the patient and family.

58. There are consistent suggestions that adaptive and assistive devices should be used for safety and function, if other methods of performing the task/activity are not available or cannot be learned or if the patient’s safety is a concern.

60. There are consistent strong recommendations that Discharge Planning should include all members of the multidisciplinary team, and the patient and family.

61. There are consistent strong recommendations that Discharge Planning should articulate patient and family circumstances.

65. There are consistent suggestions that every member of the multidisciplinary team should take responsibility for planning and monitoring the continuation of care.

67. There are consistent strong recommendations that information about patient progress should be recorded formally in patient notes and shared at Discharge Planning meetings.

68. There are consistent suggestions that one member of the multidisciplinary team should take overall responsibility of Discharge Planning to ensure continuity.

74. There are consistent suggestions that longer term care for stroke survivors should reflect their goals and circumstances.

**Step-wise approach**

The Grimshaw steps [23] provided us with easy-to-understand, and acceptable, guidance. They supported us to work in the relatively unchartered territory of determining the relevance of the international evidence base to local South African contexts, and establishing actionable implementation plans that addressed real barriers (which often differed between settings) [6, 9–15, 21, 22].

- Prepare well: The composite evidence base, derived from the 16 stroke rehabilitation CPGs, was welcomed by the team, which recognised that it did not have to do this work prior to discussing local implementation [5, 6]. The evidence synthesis methods were explained to the attendees at each project team meeting to ensure a common understanding of the recommendation production process. This follows accepted practice in CPG development [44, 47].
- Invite the relevant people: The initial meeting was attended by approximately 40 people. Subsequent meetings were attended by a core group of approximately 15 people (reflecting all end-user groups), as well as new people who developed an interest in the project, or who deputised for others who could not attend. This was why repeated explanations of the new CPG methods was essential. Most participants attended at their own cost (or were nominally funded by their institution), as the budget for the project did not extend to travel costs, venue hire, reimbursement for time or even refreshments. Attendance at meetings often required extensive travel (8 hours driving or 2-hour flights) and overnight stays. An important feature was the regular attendance at meetings of National and Provincial rehabilitation policy-makers, who provided insights into current and future Government policy, priorities, appetite for change and funding.
- Develop a proposal for change that is evidence based, feasible and attractive: The ACA decision-making algorithm (Fig. 2), the barrier prompts (Table 1) and the ACA flowchart (Fig. 3) provided the step-by-step guidance required to ensure that discussions were focused on implementation strategies relevant to South African stroke rehabilitation settings. The efficiency of discussions and arriving at actionable solutions during meetings were important to participants, whose time was valuable and who wished to improve the quality of stroke rehabilitation with maximum impact for minimum effort.
- Study the main difficulties in achieving the change, and select a set of strategies and measures at different levels linked to that problem, within your budget and possibilities: The biggest challenges in this work were to give participants the confidence to identify and ‘unpick’ local barriers to effective evidence uptake, and to encourage them to identify practical local solutions. Given the range of South African settings in which stroke rehabilitation occurs, it was remarkable therefore that so many [18] recommendations were considered adoptable within 6 months, and did not require additional effort for contextualisation or adaptation.
- Define indicators for measurement of success and monitor progress continuously or at regular intervals: Whilst participants recognised the importance of demonstrating, using defensible data, that quality improvements were being made in delivery of equitable, accessible and effective care, the task of identifying appropriate measures has not yet been achieved. This reflects not only the lack of standard national, provincial or even regional data collection about any health condition or rehabilitation activity [5, 18], but also the need to produce a set of agreed outcomes for stroke rehabilitation that map to patient care pathways, and are relevant to all stages of stroke rehabilitation.

## Abbreviations

ACA: Adopt–Contextualise–Adapt
ADAPTE Group: The ADAPTE Collaboration is a partnership between two independent groups focusing on guideline adaptation (ADAPTE group, and the Practice Guideline Evaluation and Adaptation Cycle (PGEAC) group). There is no expansion for ADAPTE (p.49) [38, 39]
CPG: clinical practice guidelines
GRADE: Grades of Recommendation Assessment, Development and Evaluation [40, 47, 49]
GRADE-ADOLOPMENT: A new term added to the Clinical Epidemiology Lexicon [49]
HICs: high-income countries
LMICs: low- and middle-income countries
PHC: primary healthcare
SA: South Africa
UK: United Kingdom
USA: United States of America
WHO: World Health Organisation

## Authors’ information

KG is Professor Extraordinaire, Stellenbosch University, Cape Town, South Africa, and visiting Research Fellow, Flinders University, South Australia, Australia.

QL is Professor of Rehabilitation, Stellenbosch University, Cape Town, South Africa.

JD is Senior Research Fellow, University of South Australia, South Australia, Australia.

S-MB is Assistant Professor, Hong Kong Polytechnic University, Hong Kong (formerly Research Fellow, Stellenbosch University, Cape Town, South Africa).

DE is Senior Lecturer, Stellenbosch University, Cape Town, South Africa.

CW is Director, Cochrane South Africa, Cape Town, South Africa
